# Using genetic markers to unravel the origin of birds converging towards pre-migratory sites

**DOI:** 10.1038/s41598-018-26669-x

**Published:** 2018-05-29

**Authors:** Anastasios Bounas, Dimitris Tsaparis, Marco Gustin, Kresimir Mikulic, Maurizio Sarà, Georgios Kotoulas, Konstantinos Sotiropoulos

**Affiliations:** 1 Molecular Ecology and Conservation Genetics Lab, Department of Biological Applications and Technology, University of Ioannina, 45110 Ioannina, Greece; 2 Hellenic Ornithological Society – BirdLife Greece, Themistokleous 80, 10681 Athens, Greece; 3 Institute of Marine Biology, Biotechnology and Aquaculture, Hellenic Centre for Marine Research, Heraklion, 71500 Greece; 4 LIPU (Lega Italiana Protezione Uccelli) - BirdLife Italia, Conservation Department, Via Udine 3, I-43100 Parma, Italy; 5 Association BIOM – BirdLife Croatia, Preradoviceva 34, 10000 Zagreb, Croatia; 6 Section of Animal Biology, Department STEBICEF, University of Palermo, Via Archirafi 18, 90123 Palermo, Italy

## Abstract

Identifying patterns of individual movements in spatial and temporal scales can provide valuable insight into the structure of populations and the dynamics of communities and ecosystems. Especially for migrating birds, that can face a variety of unfavorable conditions along their journey, resolving movements of individuals across their annual cycle is necessary in order to design better targeted conservation strategies. Here, we studied the movements of a small migratory falcon, the Lesser Kestrel (*Falco naumanni*), by genetically assigning feathers from individuals of unknown origin that concentrate in large roosts during the pre-migratory period. Our findings suggest that birds from multiple breeding populations in the Central and Eastern Mediterranean region move towards two pre-migratory sites in the Balkans, some of them detouring greatly from their expected flyways, travelling more than 500 km to reach these sites and prepare for the post-nuptial migration. By identifying the origin of individuals using the pre-migratory sites, not only we provide a better understanding of the possible impact of local threats at these sites on multiple breeding populations but also inform the design of effective conservation actions for the species.

## Introduction

Identifying patterns of individual movements in spatial and temporal scales (i.e. migration and dispersal), can provide insights into the structure of populations and the dynamics of communities and ecosystems^1–3^ and to this aim, a wide variety of markers, both extrinsic and intrinsic, have been developed for different organisms^4–6^. Specifically for birds, extrinsic markers such as ringing and tracking devices have undoubtedly offered a great amount of information regarding routes, strategies, and mechanisms of migration as well as mortality and dispersal of individuals building towards more effective conservation strategies for migratory birds^7–11^. Despite this wealth of information, such methods do have limitations mainly due to the need of recovering the individuals to obtain the desired data as, typically, return rates are low^12^. Although satellite tracking does not require recapturing the individuals, it can however be applied only on relatively large birds due to weight restrictions, while it can come at great cost when information based on a large sample size is needed. On the other hand, intrinsic markers only require an initial capture, but in order to be informative, they need to differ at the population level while these differences have to be quantified a priori^13^. Stable isotope and genetic markers have been the most commonly used intrinsic markers which either used separately or combined, have contributed significantly to the study of bird movements during the last decades^14,15^.

The use of genetic markers is based on the premise that populations show some levels of genetic structuring. Briefly, different areas of the genome can be targeted (mtDNA, microsatellites, single nucleotide polymorphisms) and as long as these markers are either population-specific or the genetic variation shows a geographical structure on the breeding range, then any unknown sampled individual could theoretically be assigned back to their breeding populations with the use of assignment tests^16,17^. Such approach has proved to be useful in multiple issues such as wildlife poaching^18–20^, illegal translocations^21^ or even used to identify a fishing competition fraud^22^. Regarding bird migration, genetic markers have been used, mostly at larger spatial scales, in order to link species populations’ breeding and wintering grounds (i.e. migratory connectivity)^4,23^. However, only a few studies^24–26^ have addressed so far the origin of individuals gathering at staging areas or stopover sites during migration, as insufficient genetic differentiation among populations of migratory species can render the assignment efforts futile^27^. Since migrating birds are subject to a number of unfavorable conditions that could, in turn, have an effect at a breeding population-level^28^ resolving movements of individuals across their annual cycle is necessary in order to assess the impact of local threats to population dynamics^29^ as well as to design effective conservation strategies for migratory birds^30,31^.

The Lesser Kestrel (*Falco naumanni*) is a small migratory falcon which breeds from the Mediterranean across the Middle East and Central Asia up to Mongolia and China, while its wintering grounds are located in sub-Saharan Africa^32^. The species underwent rapid declines throughout its European range since the early 1960s, especially in Central and Eastern Europe. Local extinctions and range contraction led to a fragmented distribution of the species^33,34^. Currently, Italy and Greece host several thousand pairs accounting for the 36% of the species’ European population^35^ whereas some smaller populations exist in the Former Yugoslav Republic of Macedonia and the Turkish Thrace. A single colony located in Croatia is considered the northernmost population of the species in Europe^36^. Lesser Kestrels do not migrate directly to Africa but show a pronounced pre-migratory behaviour that can last for several weeks. That is, after the breeding period and before the post-nuptial migration, adults and juveniles move to specific areas where they form large gatherings and roost communally, mainly in trees. There they exploit the temporal abundance of food resources, mainly insects, so as to build up the appropriate fat reserves and replace – at least partially - their flight feathers (i.e. moult)^37–39^. It has also been suggested that such gatherings may facilitate the selection of future breeding habitats and aid spring navigation^40^. Therefore, the importance of pre-migratory areas has been discussed in previous studies^41,42^ for a number of different species^43–46^. Although data from tracking devices and ringing recoveries have revealed few specific movements of individuals towards known pre-migratory sites in the Iberian^37,47^, Apennine^38,48^ and the Balkan Peninsula^49^, detailed knowledge concerning the origin of individuals in pre-migratory sites is rather restricted.

**Figure 1:**
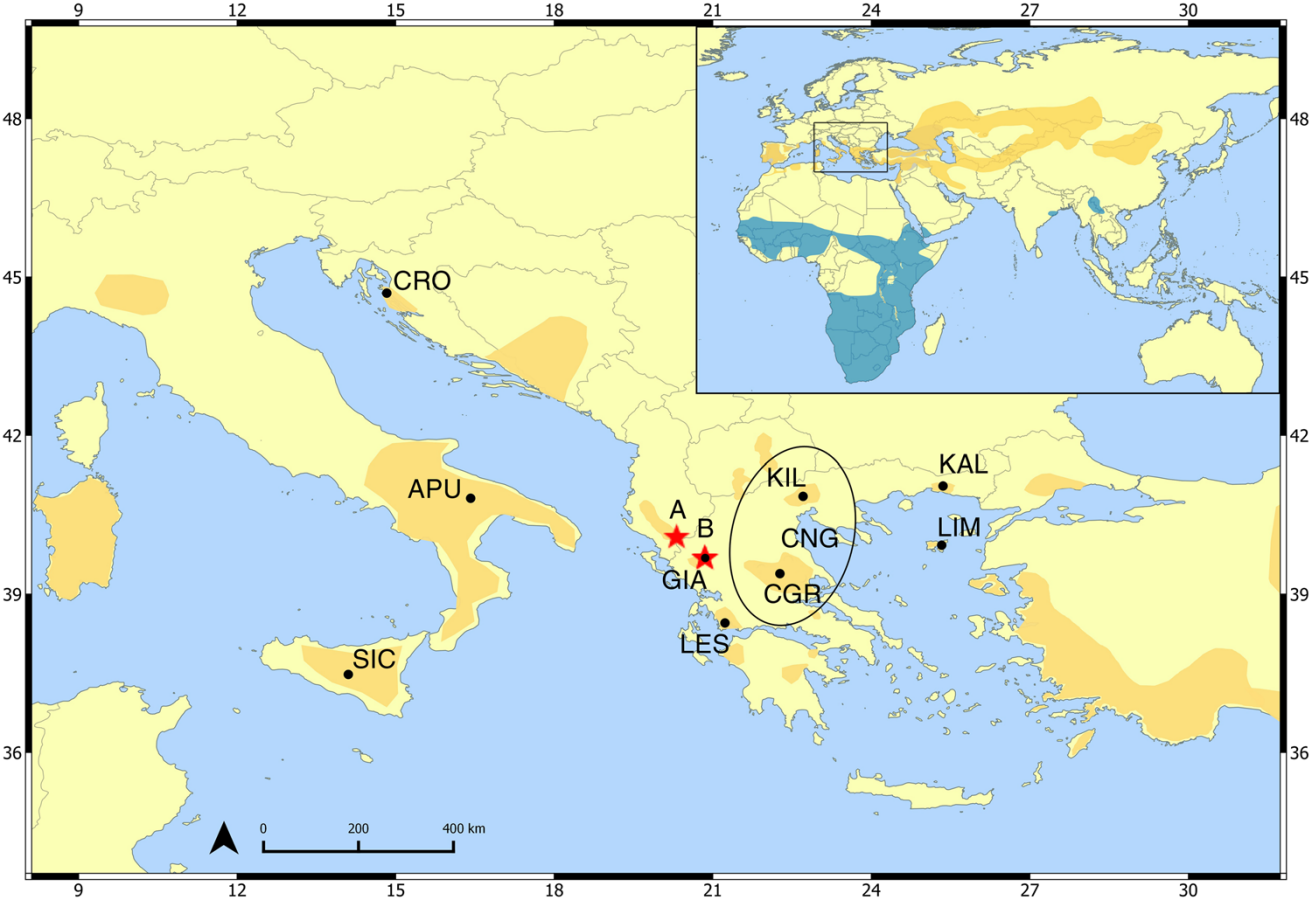
Study area and sampling locations. Red stars indicate the two pre-migratory sites, Drino valley (**A**) and Ioannina city (**B**). Black dots show the sampled breeding populations. SIC: Sicily (n = 12); APU: Apulia (n = 44); CRO: Croatia (n = 14); GIA: Ioannina (n = 24); LES: Agrinio (n = 16); CGR: Central Greece (n = 60); KIL: Kilkis (n = 13); KAL: Komotini (n = 20); LIM: Limnos Island (n = 11). Yellow and blue shaded areas represent the breeding and wintering distribution of the Lesser Kestrel respectively (modified from BirdLife International). Locations pooled for the purposes of the analyses are circled (CNG: Central-North Greece). Map was generated in QGIS v.2.12.3-Lyon (www.qgis.org/en/site/)^84^.

In this study, we genetically assigned moulted Lesser Kestrel feathers collected from the two largest pre-migratory gatherings in the Balkan Peninsula: Drino Valley in Albania, hosting a minimum of 4,000 individuals^50^ and Ioannina city in Greece, with almost 2,500 birds congregating annually^51^. For assignment of feathers to putative populations of origin, we additionally sampled nine breeding sites in the adjacent regions (Fig. 1) and implemented three different assignment methods using microsatellite genotype data. The main aim of this study is to investigate the origin of individuals gathering at the pre-migratory sites and subsequently reconstruct the pre-migratory movements of Lesser Kestrels in the Central and Eastern Mediterranean region. The results contribute to understanding the magnitude of the impact of local threats at stopover sites providing key information for the design of effective conservation actions for the species.

## Results

**Figure 2:**
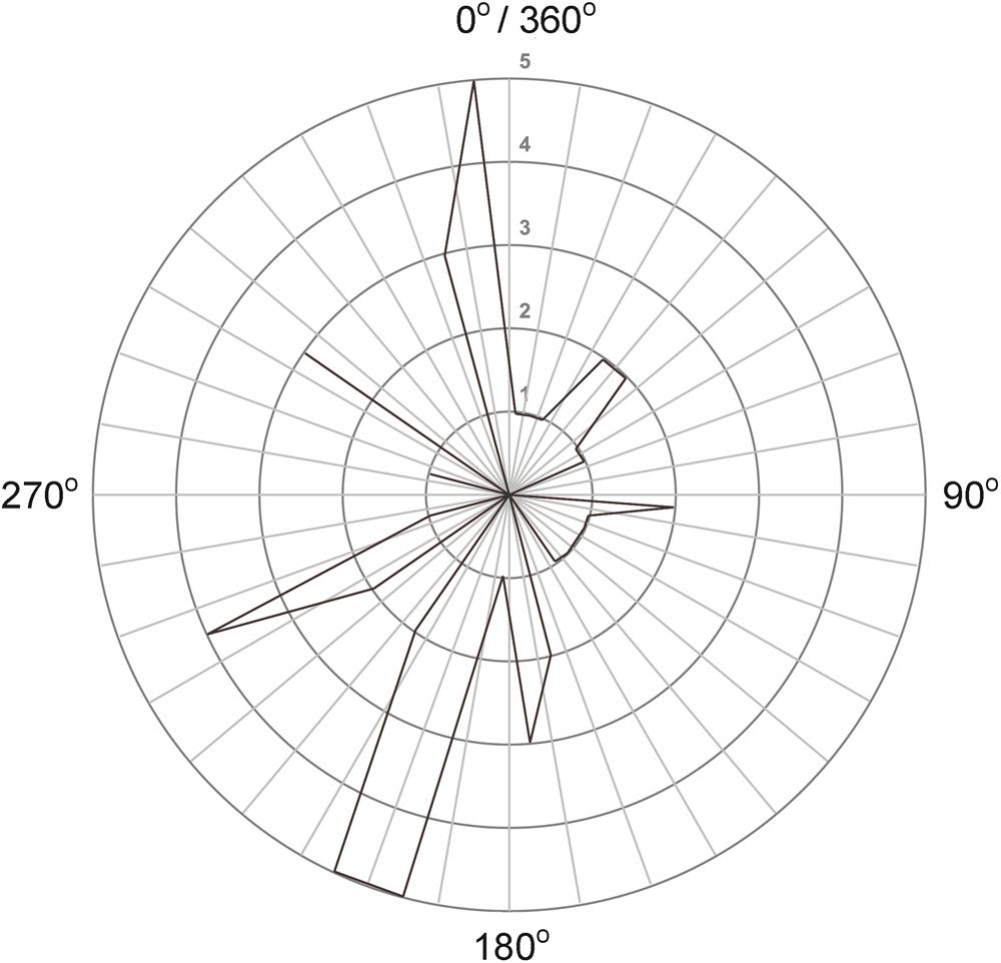
Absolute frequency of significant allele frequency clines. The graph summarizes all statistically significant associations (p < 0.05) between the allele frequencies (for 16 microsatellite loci) and the coordinates of the nine reference populations. Orientation is given with respect to the 360 degrees virtual rotating axis and all clines are distributed in arches of 10 degrees for graphical purposes. The figure was generated in GenoCline v1.0. (http://www.didac.ehu.es/genocline/)^81^.

Multilocus genotypes of 146 moulted feathers from Lesser Kestrels of unknown origin were used in the analysis. 116 of them were collected from the Ioannina roost whereas 30 were sampled from the Drino Valley roost. Reference populations showed a significant spatially patterned distribution of their allele frequencies on a North to South-west axis (Fig. 2), allowing us to attempt the assignment of the unknown individuals. Using both the partial and the fully Bayesian assignment methods implemented in GeneClass2^52–54^ and Structure^55^ respectively, we were able to confidently assign (Structure membership coefficient q > 0.70 and GeneClass2 score >90), 31 out of 146 unknown individuals (21.2%) to a reference population. The majority (19) of these 31 individuals were assigned to Central and Northern Greece (CNG), 6 individuals were assigned to Central Italy (APU), 4 were assigned to the local breeding population of Ioannina (GIA), whereas Agrinio (LES) and Komotini (KAL) had a single individual assigned to each one of them (Supplementary Table 1). No Lesser Kestrels were assigned to Croatia (CRO), Sicily (SIC) or Limnos Island (LIM).

**Figure 3:**
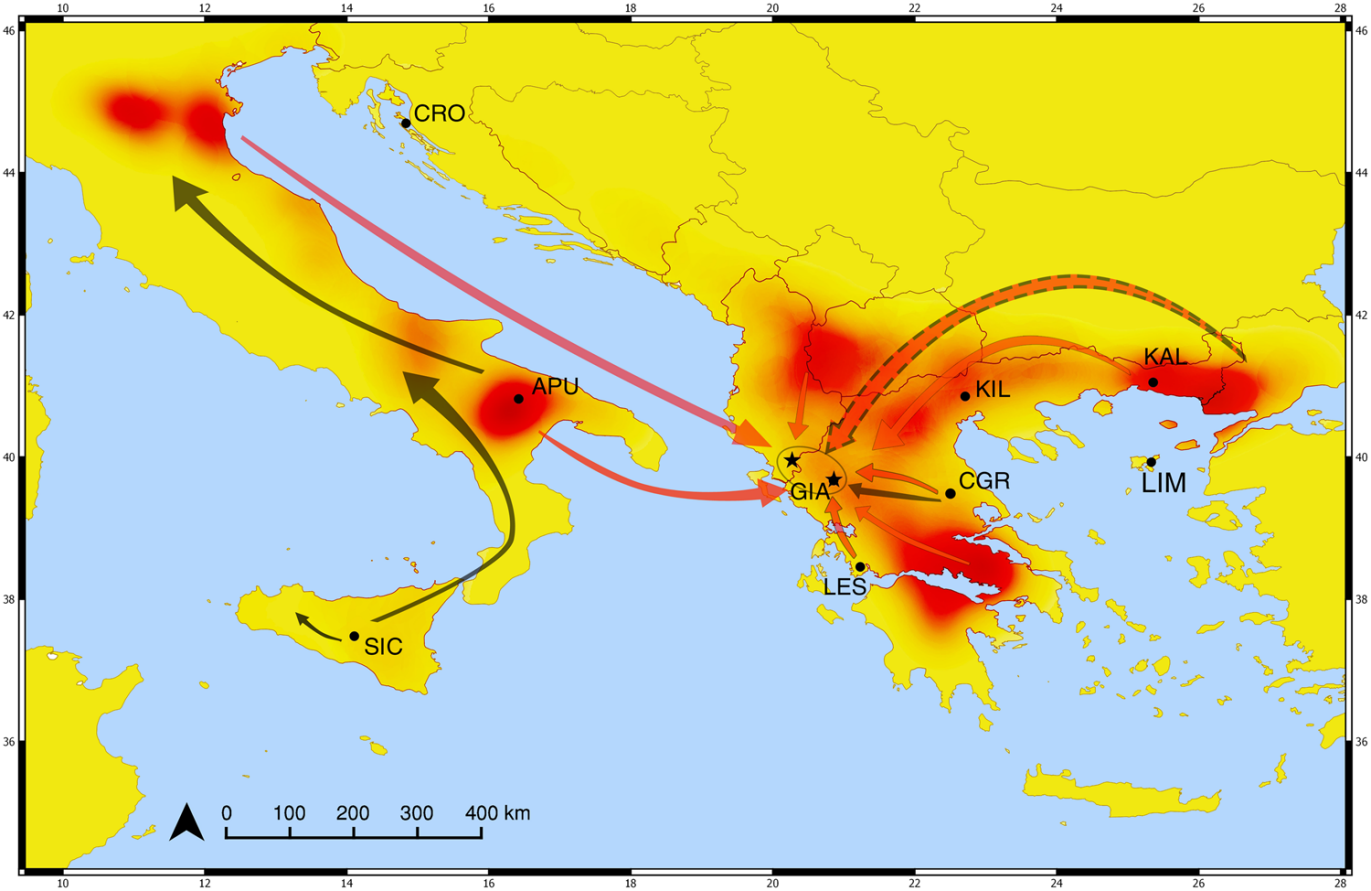
Reconstruction of pre-migratory movements of Lesser Kestrels in Central and Eastern Mediterranean. Red (burnt) areas show locations of origin as provided by the continuous assignment method. Red arrows show the inferred directions of movements towards the pre-migratory sites (black stars). Black arrows indicate movements reported by ringing recoveries^38,48,49^. Dashed arrow shows the putative movement of individuals through areas with increased presence of migrating Lesser Kestrels^58^. Black dots correspond to sampling locations. SIC: Sicily (n = 12); APU: Apulia (n = 44); CRO: Croatia (n = 14); GIA: Ioannina (n = 24); LES: Agrinio (n = 16); CGR: Central Greece (n = 60); KIL: Kilkis (n = 13); KAL: Komotini (n = 20); LIM: Limnos Island (n = 11). Map was generated in QGIS v.2.12.3-Lyon (www.qgis.org/en/site/)^84^.

SCAT assigned with confidence a total of 55 unknown individuals (37.7%), where the assignment of 31 of them was in agreement with the previous methods. Nevertheless, the spatial smoothing method implemented by SCAT was able to make the distinction between Central (CGR) and a Northern Greek population (KIL) and further assigned five individuals (Supplementary Table 1) to the reference populations of Apulia (APU), Komotini (KAL), Ioannina (GIA) and Kilkis (KIL). All individuals that were assigned to a reference population by all three assignment tests are presented in Table 1. The continuous assignment algorithm was able to provide putative (i.e. unsampled) locations of origin for 19 additional individuals (Fig. 3, Supplementary Table 2).

**Table 1:** Summarizing table of all individuals of unknown origin assigned to a reference population by three assignment tests.

Sampling location	N	Year	Reference populations										
			SIC	APU	CRO	GIA	LES	CGR	KIL	KAL	LIM	unresolved	
												n	%
Ioannina City	52	2013	0	3	0	1	1	4	2	1	0	40	76.9
	40	2014	0	1	0	2	0	2	2	0	0	33	82.5
	24	2015	0	0	0	0	0	4	0	0	0	20	83.3
Drino valley	30	2016	0	2	0	1	0	2	3	0	0	22	73.3
Total	146		0	6	0	4	1	12	7	1	0	115	78.8

N = number of feathers analysed. SIC: Sicily; APU: Apulia; CRO: Croatia; GIA: Ioannina; LES: Agrinio; CGR: Central Greece; KIL: Kilkis; KAL: Komotini; LIM: Limnos Island.

Finally, self-assignment tests that were used to assess the accuracy of each assignment method showed that SCAT performed better than any other method (92.8% correct assignments) followed by Structure (89.6%) and GeneClass2 (58.5%) (Supplementary Tables 3–5).

## Discussion

Our results show that Lesser Kestrels gathering at the two pre-migratory roosts in the Balkans potentially originate from multiple populations across the species’ breeding range, and some of them perform long-distance movements towards these sites. Such long-distance or even northward movements identified in this study could be considered an unnecessary or sub-optimal detour for migrating birds^56^. However, food availability, and specifically grasshopper abundance, is higher in mountainous areas during the summer^38,39,57^ and can subsequently act as a determinant of pre-migratory movements in the region. Meanwhile, before ending up in the roosts, birds could also exploit habitats on the move thus accounting for such transport costs. For example, birds originating from Northern Greece or potentially from Turkish Thrace, might have initially passed through highlands in Bulgaria, as numerous observations of individuals have been made after the breeding season in the country, despite the absence of any known large gatherings^58^. This way birds could take advantage of the temporal high availability of prey before making their way to the pre-migratory roosts (Fig. 3).

We should also consider any possible implications of our results in the genetic structure of the species in the region. Besides, the genetic structure of long-distance migrants cannot be unambiguously defined based solely on geography and life stage^59^ but can be shaped by other factors such as non-breeding distributions^60^. According to the post-fledging exploration hypothesis^61^, young birds disperse before migration in search of favorable areas that could be selected as their future breeding sites. In this study we found that Ioannina pre-migratory site hosts individuals originating from Italy and Central Greece, two of the populations that exhibit high levels of gene flow only with Ioannina breeding population^34^. Therefore, some of the juvenile birds visiting the Ioannina roost could return to breed in the city, thus such pre-migratory movements could influence the breeding population structure in the region.

One of the most intriguing results of this study is the detection of putative areas of origin inferred by the continuous assignment method. The majority of putative areas coincide with already known breeding sites of the species such as northern Italy, F.Y.R.O.M., Turkish Thrace as well as smaller colonies in southern Greece. Although some variability in the geographic estimates is expected, the breeding status of the species is still rather precarious in Albania and Montenegro, so more intensive fieldwork in these areas could reveal breeding locations that are suggested by our results. Apart from the located populations of origin, we did not find any sign of birds originating from Croatia, Sicily or Limnos Island. A plausible explanation could be the existence of differences in the breeding phenology and/or variations in the pre-migratory and migratory strategy of these populations: Croatian Lesser Kestrels fledge in late July and move locally before migration^36^ whereas most individuals from Sicily perform intra-island movements thus finding enough suitable feeding grounds during this period^38^. However, although not detected by our analyses, the possibility that some individuals from other breeding populations may actually visit both pre-migratory areas in the Balkans should not be ruled out. Sheer chance (i.e. failure to sample any feathers belonging to birds from these populations) or different moulting strategies^62^ (i.e. some birds might not moult in the pre-migratory sites but rather perform a complete moult in their wintering grounds) could be further possible explanations.

Our results conform to the expected elevated performance of the spatial smoothing method in relation to other assignment methods, especially when applied at a regional level^63^ and when the underlying structure of populations is shaped from isolation by distance processes. Unlike SCAT, most other standard assignment methods suffer from two serious drawbacks. Firstly, they can have a strong bias against the assignment of samples to reference populations of smaller sample sizes because reduced sample size can give inaccurate allele frequencies^20^. Secondly, they both assume that the true population of origin has been sampled^18,54^. The methods implemented in SCAT overcome these limitations of the standard assignment tests. However, our success rates were not high as SCAT was able to assign only 55 out of 146 unknown individuals (37.7%) whereas the other two methods performed less well (21.2%) although results were within the range of other spatial assignment studies^63^. Low assignment rates in assignment studies can be explained by the ecology and behaviour of the focal species, as patterns of genetic differentiation and gene flow are highly influenced by habitat characteristics and migratory behaviour^64^. Migratory populations of raptors have been found to generally have weaker genetic structure than non-migratory ones^64,65^, because for example some individuals might migrate through a different route upon their return on the breeding grounds, and consequently disperse and breed far from their natal site^66^. In fact the genetic structure among Lesser Kestrel populations is relatively weak^34^, therefore some genotypes can be relatively common in more than one potential source populations and unassigned individuals could possess shared common alleles and no rare alleles at some loci, thus showing probabilistic membership to multiple populations. However, the presence of private or rare alleles in the populations along with the spatially patterned distribution of their allele frequencies enabled the assignment of some individuals. Additionally, the number of samples genotyped and our marker of choice may be a limitation on our assignment inference. More training samples or the use of SNPs could improve the assignment accuracy^63^.

Despite that, our results are still relevant under a conservation perspective. Both pre-migratory sites should be considered as a single target area for the implementation of conservation actions for the species, since the distance between them is short (approximately 60 km “as the crow flies”) and they host individuals from the same breeding areas. Thus, individuals could easily move between the two sites. The pre-migration period could influence the survival of individuals since birds optimize their condition for migration. As Lesser Kestrels are highly gregarious during this period, even small scale threats can have a great impact on a large number of birds^31^. It has been reported for example that the mountainous areas adjacent to Ioannina city play a major role as foraging grounds for the Lesser Kestrel during the premigratory period^39^ thus any habitat degradation could compromise birds during this critical stage of their annual cycle. Resolving the pre-migratory movements in Central and Eastern Mediterranean region will help to understand the magnitude of local-threat impacts on the species and highlight the importance of these stopover sites for breeding populations that span across different countries.

## Methods

### Sample collection

Moulted feathers of Lesser Kestrels were collected from the pre-migratory roost of Ioannina city over a 3 year period (2013–2015) and the pre-migratory roost of Drino Valley in 2016 (Fig. 1). The non-invasive sampling of feathers took place weekly from the second week of July until the second week of September to cover the whole range of the pre-migratory period and samples were stored separately in paper envelopes until DNA extraction. To minimize the probability of sampling more than one feather from the same individual, feathers were aligned and selected for extraction based on their external features (colour, pattern). We also recorded the age class of the individual (adult, juvenile) based on visual inspection of the feather when possible. To assign the unknown individuals to a source of origin, samples from nine breeding populations in Central and Eastern Mediterranean were obtained (Fig. 1). During the 2013 to 2016 breeding seasons, two drops of blood (≈50 µl) from 214 unrelated individuals were obtained by leg-pricking and stored in blood storage cards (NucleoCards®) at room temperature until DNA extraction. This study was carried out in accordance with the European Convention for the Protection of Vertebrate Animals Used for Experimental and Other Scientific Purposes of the Council of Europe (http://conventions.coe.int/Treaty/EN/Treaties/Html/123.htm.). Sampling was approved by every relevant national authority: University of Ioannina and the Hellenic Ministry of Environment and Energy (130551/1277) in Greece, University of Bari and Instituto Superiore per la Ricerca e la Protezione Ambientale-ISPRA (n. 0020267) in Italy, and the Croatian Ministry of Environment (KL: UP/I-612-07/15-48/108; URBROJ: 517-07-1-1-1-15-3).

### Amplification and Genotyping

DNA from a total of 163 flight and tail feathers was extracted using both the basal tip of the calamus and the blood clot from the superior umbilicus of each feather sample^67^ using NucleoSpin Tissue kit (Macherey-Nagel). In cases of feathers without or a barely visible blood clot, we performed a manual extraction following an improved method for moulted feathers^68^. DNA from blood samples was extracted using the NucleoSpin Tissue kit (Macherey-Nagel) following the manufacturers’ protocol. All individuals were genotyped at 16 microsatellite loci (Fp5, Fp31, Fp46-1, Fp79-4, Fp86-2, Fp89, Cl347, Fnd1.3, Fnd1.4, Fnd1.6, Fnd1.7, Fnd1.8, Fnd2.3, Fnd2.4, Fnd2.6, Fn1-11)^69–72^. Procedures on loci amplification and their properties are described in detail in Bounas *et al*.^34^. Reliable genotypes from feather samples were obtained using the multiple tubes approach^73,74^ and consensus genotypes were retrieved using GIMLET^75^. Due to this approach, it was possible to calculate error rates in feather amplification, by counting directly the wrong genotypes in relation to the total number of genotypes retrieved^76^ across all loci. We used the package “MsatAllele”^77^ in R, version 3.2.2^78^ to allocate the alleles to their respective size classes. Genotyping errors due to null alleles and stuttering were examined for each locus and reference population using Micro-Checker^79^. Deviations from Hardy-Weinberg proportions (HWE) at each locus and reference population as well as linkage disequilibrium (LD) between pairs of loci were tested in Genepop 4.2^80^ following program’s default settings. The results of the above analyses are given in the Supplementary Results. Finally, we used GIMLET to check for duplicate genotypes in feathers collected each year to further avoid sampling the same individuals. Only feather samples that showed a unique genotype and amplified in more than 12 loci (n = 146) were used in further analysis.

### Data analysis and assignment tests

We examined any spatial patterns of allele frequencies in the reference populations using the software Genocline v.1.0^81^ (http://www.didac.ehu.es/genocline/). The method calculates the correlation coefficient between the frequency of each allele (after angular transformation) and the populations’ spatial location with respect to an axis that rotates 360 degrees in consecutive iterations of one degree. A statistically significant association (p < 0.05) will be indicative of a spatially patterned distribution of the allele frequencies. Feathers of unknown origin were then assigned to a reference population using a combination of the probabilities from the Bayesian method^53^ implemented in GeneClass2 and the membership coefficients (q) from the analysis in Structure. The sampled population of Central Greece (CGR) and the population of Kilkis (KIL) were merged together (CNG) as they were not found to be significantly differentiated and assignment attempts would be inconclusive^34^. All other sampled populations were considered as different sources, thus origin assignments were based on a total of eight reference populations. An individual was considered to be of resolved origin if the corresponding score in GeneClass2 was greater than 90 and the Structure membership coefficient was above 0.70. Those thresholds were chosen after comparison with other assignment approaches^16,82^. In cases where individuals did not meet the above criteria or the assignment result of the two methods disagreed, they were considered unassigned. For Structure analysis we considered the origin of individuals to be known (except the ones to be assigned), allele frequencies were updated using only the individuals of known origin and assumed admixture among reference populations^18^. Runs were set with a burn-in period of 2 × 10^5^ iterations followed by 10^6^ MCMC steps with 20 replicates for a fixed value of K = 8.

The Smoothed and Continuous Assignments (SCAT) analysis uses a Bayesian method implemented with MCMC spatial smoothing^20,83^ in order to estimate allele frequencies at any location depending on all reference samples and the distance between them. This method seemed to be ideal for our case since it is based on the premise that allele frequencies show some spatial patterning, which is the case in our reference populations (Fig. 2). Therefore, each of our nine sampled populations was considered as a reference one. We adopted the strategy followed by Wasser *et al*.^19^ to continuously assign individuals of unknown origin: we performed three initial runs, without assignments (i.e. without –A option), using a relatively large number of iterations (Niter = 100, Nthin = 1000, Nburn = 100) in order to get estimates of the parameters α and β. Then we fixed the parameters to their estimated modal values (α_0_ = 0.5, α_1_ = 5,087, α_2_ = 0.86, β = 3.55) and performed five independent assignment runs, each one starting with a different seed for the pseudorandom number generator. For each run we discarded the first 2,000 iterations as burn-in, and stored every 10^th^ of the following 1,000 iterations, resulting in 100 putative locations of origin for each sample. The range of allowable locations for the continuous assignment of individuals was specified by creating a boundary file of the contemporary extent of Lesser Kestrel distribution in the Central and Eastern Mediterranean and Middle East. Boundaries linked all discontinuous range but did not include open sea regions. The uncertainty of each assignment was assessed by examining the density of the plausible location points. If location points clustered tightly together in a single source of origin the individual was considered to be assigned with high confidence, whereas putative location points spreading all over the landscape or concentrated in two or more areas showed high uncertainty and the individuals were left unassigned. The spread of points and plausible locations were assessed and visualized using the heatmap plugin in QGIS v.2.12.3. As an error measurement we calculated the standard distance of the median coordinates for each assignment. The concentration of points around the median coordinates can then be represented as circle with a radius equal to the standard distance value. SCAT v.2.1 was compiled from the source code available at https://github.com/stephens999/scat. Finally, the assignment accuracy of each method was evaluated by performing self-assignment tests on the reference populations (each individual was assigned to its own and all other reference populations) and the average accuracy for each method was calculated (proportions of correctly assigned individuals within population sets).

### Data availability

The raw genotype dataset generated and analysed during the current study (DatasetLK) is included in this published article (and its Supplementary Information files).

## Electronic supplementary material

Supplementary information

Supplementary DatasetLK
