# Supplementary material for: Using genetic markers to unravel the origin of birds converging towards pre-migratory sites
Source: Sci Rep. 2018 May 29;8:8326. doi: 10.1038/s41598-018-26669-x (PMC5974135; doi:10.1038/s41598-018-26669-x)
Supplement: Supplementary file 1 — Supplementary information [file 41598_2018_26669_MOESM1_ESM.docx]

**Supplementary Information**

**Using genetic markers to unravel the origin of birds converging towards pre-migratory sites**

Anastasios Bounas^1,2*^, Dimitris Tsaparis^3^, Marco Gustin^4^, Kresimir Mikulic^5^, Maurizio Sarà^6^, Georgios Kotoulas^3^, Konstantinos Sotiropoulos^1^

^1^Molecular Ecology and Conservation Genetics Lab, Department of Biological Applications and Technology, University of Ioannina, 45110, Ioannina, Greece.

^2^Hellenic Ornithological Society – BirdLife Greece, Themistokleous 80, 10681 Athens, Greece.

^3^ Institute of Marine Biology, Biotechnology and Aquaculture, Hellenic Centre for Marine Research, Heraklion, 71500, Greece.

^4^LIPU (Lega Italiana Protezione Uccelli) - BirdLife Italia, Conservation Department, Via Udine 3, I-43100, Parma, Italy.

^5^Association BIOM – BirdLife Croatia, Preradoviceva 34, 10000, Zagreb, Croatia.

^6^ Section of Animal Biology, Department STEBICEF, University of Palermo, Via Archirafi 18, 90123 Palermo, Italy.

^*^Corresponding author: [abounas@cc.uoi.gr](mailto:abounas@cc.uoi.gr)

**Supplementary Results**

On average, loci and reference populations conformed to HW proportions but some deviations were detected: the Croatian population (CRO) deviated at two out of the 16 loci (Fp31, Fp86-2). Since these loci did not show consistent deviations across all populations, we included them in subsequent analyses attributing this disequilibrium in processes specific to those populations. No linkage disequilibrium was detected between any pair of loci across all populations, genotyping error rates for feather samples were low (mean=0.012; range=0-0.03) but even so, they were mitigated using the multiple tubes approach^1,2^ and no loci was found prone to null alleles. Although assignment methods assume HWE within populations, slight deviations were found to have little effect on the performance of assignment tests^3^ whereas even when loci are affected by null alleles would not significantly alter the assignment results^4^. Therefore it is unlikely that our results are biased by errors associated with microsatellite properties and scoring.

**Table S1.** List of all feather samples assigned to a reference population. Location codes correspond to those in Figure 1. Samples numbered 32-36 were only assigned by SCAT, but indicative values of Structure membership coefficient (q) and GeneClass2 score are provided.

| No | Sample ID | Collection date | Roost | Age class | Inferred origin | Structure (q) | Geneclass (likelihood) | SCAT |
| --- | --- | --- | --- | --- | --- | --- | --- | --- |
| 1 | A12 | 18/7/2016 | Drino | adult | CNG | 0.84 | 98.1 | KIL |
| 2 | A17 | 18/7/2016 | Drino | adult | CNG | 0.84 | 92.1 | CGR |
| 3 | A19 | 18/7/2016 | Drino | unknown | CNG | 0.72 | 97.2 | CGR |
| 4 | A21 | 18/7/2016 | Drino | adult | CNG | 0.89 | 90.9 | CGR |
| 5 | A24 | 18/7/2016 | Drino | unknown | APU | 0.78 | 94.2 | APU |
| 6 | A26 | 18/7/2016 | Drino | adult | GIA | 0.72 | 90.3 | GIA |
| 7 | A35 | 18/7/2016 | Drino | unknown | APU | 0.85 | 99.0 | APU |
| 8 | A40 | 18/7/2016 | Drino | juvenile | CNG | 0.87 | 99.9 | CGR |
| 9 | 106 | 22/8/2015 | Ioannina | adult | CNG | 0.74 | 97.7 | CGR |
| 10 | 120 | 24/7/2015 | Ioannina | adult | CNG | 0.88 | 98.0 | CGR |
| 11 | 125 | 31/7/2015 | Ioannina | unknown | CNG | 0.91 | 95.5 | CGR |
| 12 | 132 | 7/8/2015 | Ioannina | unknown | CNG | 0.87 | 99.9 | KIL |
| 13 | 12 | 24/7/2013 | Ioannina | juvenile | CNG | 0.89 | 99.2 | CGR |
| 14 | 16 | 30/7/2013 | Ioannina | adult | LES | 0.72 | 98.1 | LES |
| 15 | 20 | 21/8/2013 | Ioannina | juvenile | APU | 0.77 | 97.6 | APU |
| 16 | 21 | 21/8/2013 | Ioannina | adult | APU | 0.71 | 97.0 | APU |
| 17 | 26 | 13/8/2013 | Ioannina | juvenile | CNG | 0.88 | 99.4 | CGR |
| 18 | 29 | 30/7/2013 | Ioannina | adult | APU | 0.88 | 100.0 | APU |
| 19 | 31 | 30/7/2013 | Ioannina | unknown | GIA | 0.74 | 97.5 | GIA |
| 20 | 39 | 6/8/2013 | Ioannina | adult | CNG | 0.94 | 93.8 | CGR |
| 21 | 42 | 21/8/2013 | Ioannina | juvenile | CNG | 0.80 | 94.7 | CGR |
| 22 | 48 | 21/8/2013 | Ioannina | juvenile | KAL | 0.71 | 95.7 | KAL |
| 23 | 50 | 13/8/2013 | Ioannina | unknown | CNG | 0.84 | 93.7 | CGR |
| 24 | 57 | 22/8/2014 | Ioannina | juvenile | GIA | 0.71 | 94.9 | GIA |
| 25 | 59 | 22/8/2014 | Ioannina | unknown | GIA | 0.94 | 99.9 | GIA |
| 26 | 60 | 10/9/2014 | Ioannina | adult | CNG | 0.82 | 98.0 | CGR |
| 27 | 68 | 22/7/2014 | Ioannina | juvenile | CNG | 0.95 | 90.8 | CGR |
| 28 | 71 | 10/8/2014 | Ioannina | juvenile | CNG | 0.84 | 96.3 | KIL |
| 29 | 83 | 13/8/2014 | Ioannina | unknown | CNG | 0.86 | 99.8 | KIL |
| 30 | F08 | 24/7/2013 | Ioannina | juvenile | CNG | 0.91 | 99.5 | CGR |
| 31 | 97 | 26/8/2014 | Ioannina | unknown | APU | 0.73 | 99.9 | APU |
| 32 | 30 | 30/7/2013 | Ioannina | adult |  | 0.62 | 72.2 | GIA |
| 33 | A30 | 18/7/2016 | Drino | juvenile |  | 0.59 | 40.6 | KAL |
| 34 | 67 | 5/7/2014 | Ioannina | juvenile |  | 0.91 | 78.2 | KIL |
| 35 | 128 | 7/8/2015 | Ioannina | adult |  | 0.55 | 46.2 | GIA |
| 36 | A31 | 18/7/2016 | Drino | adult |  | 0.72 | 82.8 | APU |

**Table S2.** List of samples continuously assigned by SCAT along with median coordinates and standard distance.

| No | Sample ID | Roost | | Location of origin | Latitude | | | Longitude | | Standard distance (km) | |
| --- | --- | --- | --- | --- | --- | --- | --- | --- | --- | --- | --- |
| 1 | A23 | Drino | South Greece | | | 38.46 | 22.98 | | 224.9 | |  |
| 2 | A27 | Drino | Turkish Thrace | | | 41.33 | 26.66 | | 309 | |  |
| 3 | A34 | Drino | Northern Greece | | | 41.03 | 22.99 | | 169.2 | |  |
| 4 | A37 | Drino | Thrace-Turkey | | | 40.81 | 26.21 | | 122.5 | |  |
| 5 | 32 | Ioannina | South Greece | | | 38.5 | 21.69 | | 208 | |  |
| 6 | 63 | Ioannina | Albania | | | 40.26 | 20.52 | | 161.4 | |  |
| 7 | 64 | Ioannina | South Italy | | | 40.98 | 16.66 | | 203.7 | |  |
| 8 | 69 | Ioannina | Albania-FYROM | | | 41.55 | 20.64 | | 231.5 | |  |
| 9 | 76 | Ioannina | North Italy | | | 44.76 | 11.93 | | 224.9 | |  |
| 10 | 87 | Ioannina | Thrace-Turkey | | | 41.17 | 25.36 | | 149.2 | |  |
| 11 | 95 | Ioannina | Nortwestern Greece-FYROM | | | 40.59 | 21.19 | | 184.8 | |  |
| 12 | 110 | Ioannina | Italy | | | 40.57 | 16.31 | | 224.9 | |  |
| 13 | 114 | Ioannina | Northern Greece-FYROM | | | 40.91 | 23.12 | | 222.7 | |  |
| 14 | 115 | Ioannina | North Italy | | | 44.82 | 11.22 | | 305 | |  |
| 15 | 130 | Ioannina | Thrace-Turkey | | | 41.15 | 25.55 | | 171.4 | |  |
| 16 | 105 | Ioannina | Albania-FYROM | | | 41.34 | 21.22 | | 333.9 | |  |
| 17 | 118 | Ioannina | Thrace-Turkey | | | 40.95 | 26.21 | | 284.8 | |  |
| 18 | 119 | Ioannina | Peloponese | | | 37.86 | 22.72 | | 184.8 | |  |
| 19 | 131 | Ioannina | South Greece | | | 38.46 | 22.75 | | 276 | |  |

**Table S3.** Self-assignment matrix using SCAT2. Accuracy is expressed as percentage of individuals correctly assigned to their own populations. For population codes see Figure 1.

|  | Reference population | | | | | | | | |  |
| --- | --- | --- | --- | --- | --- | --- | --- | --- | --- | --- |
|  | APU | SIC | CRO | GIA | LES | CGR | KIL | KAL | LIM | Accuracy % |
| APU | 40 | 1 | 2 | 1 | 0 | 0 | 0 | 0 | 0 | 90.9 |
| SIC | 0 | 12 | 0 | 0 | 0 | 0 | 0 | 0 | 0 | 100 |
| CRO | 0 | 0 | 14 | 0 | 0 | 0 | 0 | 0 | 0 | 100 |
| GIA | 1 | 0 | 0 | 22 | 1 | 0 | 0 | 0 | 0 | 91.7 |
| LES | 0 | 0 | 0 | 1 | 15 | 0 | 0 | 0 | 0 | 93.8 |
| CGR | 0 | 0 | 0 | 2 | 2 | 48 | 5 | 2 | 1 | 80 |
| KIL | 0 | 0 | 0 | 1 | 0 | 1 | 11 | 0 | 0 | 84.6 |
| KAL | 0 | 0 | 0 | 0 | 0 | 1 | 0 | 19 | 0 | 95 |
| LIM | 0 | 0 | 0 | 0 | 0 | 0 | 0 | 0 | 11 | 100 |
|  | Average accuracy 92.8% | | | | | | | | |  |

**Table S4.** Self-assignment matrix using Structure. Accuracy is expressed as percentage of individuals correctly assigned to their own populations. For population codes see Figure 1.

|  | Reference population | | | | | | | |  |
| --- | --- | --- | --- | --- | --- | --- | --- | --- | --- |
|  | APU | SIC | CRO | GIA | LES | CNG | KAL | LIM | Accuracy % |
| APU | 35 | 1 | 1 | 2 | 0 | 5 | 0 | 0 | 79.5 |
| SIC | 0 | 12 | 0 | 0 | 0 | 0 | 0 | 0 | 100 |
| CRO | 1 | 0 | 13 | 0 | 0 | 0 | 0 | 0 | 92.9 |
| GIA | 2 | 0 | 0 | 20 | 1 | 1 | 0 | 0 | 83.3 |
| LES | 0 | 0 | 0 | 1 | 14 | 1 | 0 | 0 | 87.5 |
| CNG | 3 | 0 | 0 | 3 | 1 | 61 | 5 | 0 | 83.6 |
| KAL | 0 | 0 | 0 | 1 | 0 | 1 | 18 | 0 | 90 |
| LIM | 0 | 0 | 0 | 0 | 0 | 0 | 0 | 11 | 100 |
|  | Average accuracy 89.6% | | | | | | | |  |

**Table S5.** Self-assignment matrix using GeneClass2. Accuracy is expressed as percentage of individuals correctly assigned to their own populations. For population codes see Figure 1.

|  | Reference population | | | | | | | |  |
| --- | --- | --- | --- | --- | --- | --- | --- | --- | --- |
|  | APU | SIC | CRO | GIA | LES | CNG | KAL | LIM | Accuracy % |
| APU | 22 | 2 | 2 | 3 | 1 | 14 | 0 | 0 | 50 |
| SIC | 2 | 7 | 0 | 0 | 0 | 3 | 0 | 0 | 58.3 |
| CRO | 4 | 1 | 9 | 0 | 0 | 0 | 0 | 0 | 64.3 |
| GIA | 4 | 0 | 0 | 12 | 2 | 5 | 1 | 0 | 50 |
| LES | 1 | 0 | 0 | 2 | 8 | 5 | 0 | 0 | 50 |
| CNG | 19 | 0 | 0 | 5 | 3 | 40 | 6 | 0 | 54.8 |
| KAL | 1 | 0 | 0 | 2 | 1 | 6 | 10 | 0 | 50 |
| LIM | 0 | 0 | 0 | 0 | 0 | 0 | 1 | 10 | 90.9 |
|  | Average accuracy 58.5% | | | | | | | |  |

**References**

1 Navidi, W., Arnheim, N. & Waterman, M. A multiple-tubes approach for accurate genotyping of very small DNA samples by using PCR: statistical considerations. *American Journal of Human Genetics* **50**, 347 (1992).

2 Taberlet, P. *et al.* Reliable genotyping of samples with very low DNA quantities using PCR. *Nucleic Acids Research* **24**, 3189-3194 (1996).

3 Cornuet, J.-M., Piry, S., Luikart, G., Estoup, A. & Solignac, M. New Methods Employing Multilocus Genotypes to Select or Exclude Populations as Origins of Individuals. *Genetics* **153**, 1989-2000 (1999).

4 Carlsson, J. Effects of microsatellite null alleles on assignment testing. *Journal of Heredity* **99**, 616-623 (2008).
